# Additive Manufacturing of 3D Anatomical Models—Review of Processes, Materials and Applications

**DOI:** 10.3390/ma16020880

**Published:** 2023-01-16

**Authors:** Magdalena Żukowska, Maryam Alsadat Rad, Filip Górski

**Affiliations:** 1 Faculty of Mechanical Engineering, Poznan University of Technology, Piotrowo 3, 61-138 Poznan, Poland; 2 School of Biomedical Engineering, Faculty of Engineering and Information Technology, University of Technology, Sydney, NSW 2007, Australia

**Keywords:** additive manufacturing, physical models, methodology, soft tissues, hard tissues, medicine

## Abstract

The methods of additive manufacturing of anatomical models are widely used in medical practice, including physician support, education and planning of treatment procedures. The aim of the review was to identify the area of additive manufacturing and the application of anatomical models, imitating both soft and hard tissue. The paper outlines the most commonly used methodologies, from medical imaging to obtaining a functional physical model. The materials used to imitate specific organs and tissues, and the related technologies used to produce, them are included. The study covers publications in English, published by the end of 2022 and included in the Scopus. The obtained results emphasise the growing popularity of the issue, especially in the areas related to the attempt to imitate soft tissues with the use of low-cost 3D printing and plastic casting techniques.

## 1. Introduction

The wide possibilities of the still developing methods of additive manufacturing have also been adapted to the needs of medicine. The ability to recreate complex and non-standard shapes allows for an individualised approach to a specific patient; this is possible both at the stage of planning the operation and the stage of educating about the existing problem, by implementing surgical tools and templates dedicated to the patient, ending with individually matched (patient specific) implants or prostheses. In addition, rapid manufacturing methods have been used in tissue engineering as an element of bioprinting using living cells [1,2]. The continuous development of additive manufacturing methods provides newer solutions in the field of the materials used, obtained textures, colours and properties of the final product. Moreover, the development and expiration of certain patents for selected technologies (e.g., FFF—Fused Filament Fabrication) resulted in an increase in the availability of rapid prototyping methods, also for less specialised entities or individual users. This also affected the prices of the devices themselves (3D printers) and the materials used in the manufacturing process. This, in turn, translates into a greater availability of functional medical components that are used in various medical disciplines, which can be divided and categorised into 5 main areas: medical models, surgical implants, surgical guides, external aids and bio-manufacturing [3].

Therefore, this review will focus primarily on the area of medical models. The aim of this study is to present and compare the methodology, material and technological selection, as well as the use of physical models imitating human soft and hard tissues for various applications; it will start with simple educational models, through pre-surgery, to inter-operative supplies, used by surgeons for better performance.

## 2. Materials and Methods

### 2.1. Paper Selection Methods

**Figure 1:**
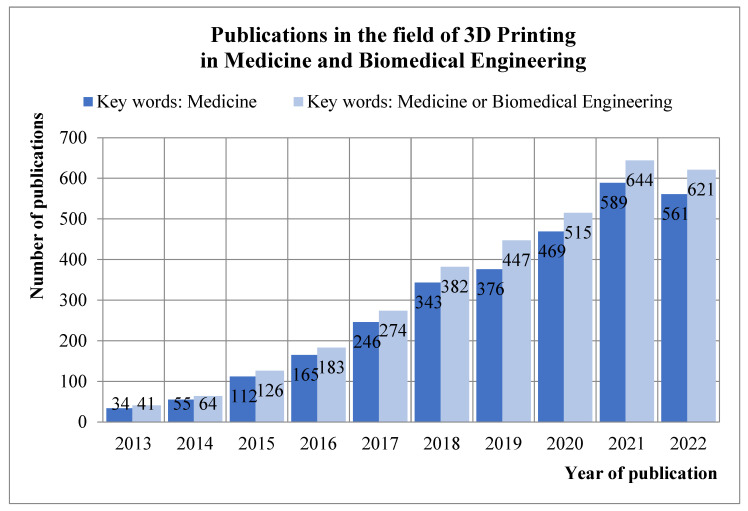
Publications on 3D printing in medicine and Biomedical Engineering published until the end of 2022 in Scopus database.

The literature selected for the review collects data from the Scopus database, published by the end of 2022 (as of 13 December 2022). The main review of the available literature in the field of 3D printing in medicine covered all types of publications (article studies, reviews, conference papers, chapters etc.), and all the available languages to be able to outline the popularity of the research topic and the frequency of publishing in this area. By entering the keywords of *“3D Printing” OR “additive manufacturing” AND medicine*, 2994 document results in the Scopus database were obtained. The beginning of a noticeable increase in published texts on this subject was found in the 2013–2015 period. The values presented in the graph (Figure 1), covering the years 2013–2022, show a clearly growing trend of publishing in the discussed subject. Because very few relevant papers were found in the period before 2013, this review was thus limited to the years 2013–2022. A significant part of the texts, as much as 93% of the articles, were published in English, while the remaining publications were successively 4% in Chinese, 1% in German and 2% collectively in other languages.

The search was performed again with broader keywords, including “biomedical engineering” OR medicine, and 2018 document results were obtained. The number of publications increased by several hundred articles, while the distribution of publications in terms of timeframe and language is comparable to the initial result.

**Figure 2:**
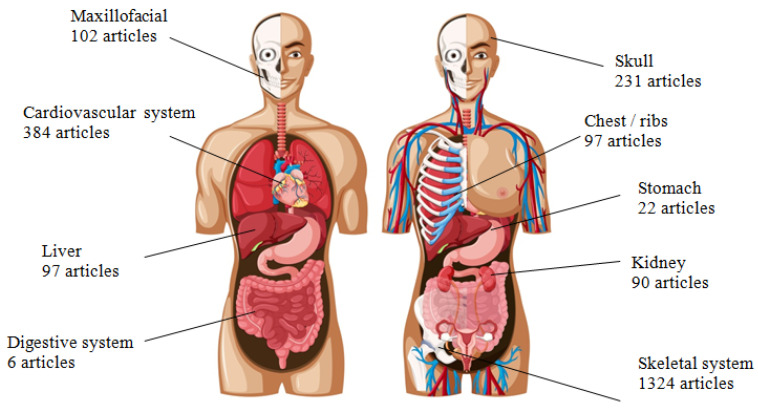
Publications on the use of physical models manufactured by 3D printing in Scopus database; divided into specific organs and systems [Image designed by brgfx/Freepik].

The aim of the review was to get acquainted with the methodology of manufacturing models and tools supporting the operational process, therefore the search was made more precise by adding the keyword “model” and excluding the word “medicine”. In addition, each consecutive search was made more specific by adding terms related to specific anatomical structures and systems. Thus, the recognition of publication and research trends in specific areas of medicine was obtained, and areas less recognised and/or studied were outlined. The searched articles were narrowed down to the “case-study” publications, published in both journals and conference books until end of 2022. The obtained results, divided into specific areas of the human body to which the selected papers apply, are shown in Figure 2. The presented searches constitute the framework for a further review of existing research, solutions, methodologies and implementations. It is important to understand the nature of the research work to distinguish the main areas in which the word “model” is used. It concerns:

- physical models for pre-operative preparation and surgery planning,
- physical models to perform simulated operations,
- physical models with a template to support the tissue reconstruction process (e.g., mandible, facial skeleton),
- surgical instruments and guiding templates to match the patient’s anatomy,
- physical models for the education and training of doctors and medical students,
- physical models for the educational purposes of the patient and his family,
- implants adjusted to the individual patient’s anatomy,
- improving the strength and quality of existing implants,
- tissue engineering and bioprinting.

Most publications about medical models produced with additive manufacturing methods concern the topic of the skeletal system. Apart from this, subgroups were additionally distinguished due to the large number of articles on single structures: the skull [4,5], facial skeleton [6,7,8], which apart from reconstructive surgery also includes dentistry and orthodontics [9,10] and the thoracic/rib region. The circulatory system, including publications concerning only the heart or other structures [11,12], and articles concerning the kidneys [13,14,15] and liver [16,17], can be classified as more recent publications. The increased frequency of publications regarding the use of models in these fields of medicine falls in the years 2016–2017. The least recognised topic is the stomach, digestive and urinary system in general, excluding the kidneys. The first publications related to these systems and organs in the Scopus database appeared in 2014–2016. In a further analysis of the literature, the review includes studies on models that imitate soft and hard tissues but are not actual tissue. Works involving research in the field of tissue engineering and bioprinting will not be included in the analysis of the methodology, material selection and the method of model evaluation, as this review is focused on aspects of the production of purely synthetic (polymeric and/or composite), not organic, models.

### 2.2. Concepts of Manufacturing

**Figure 3:**
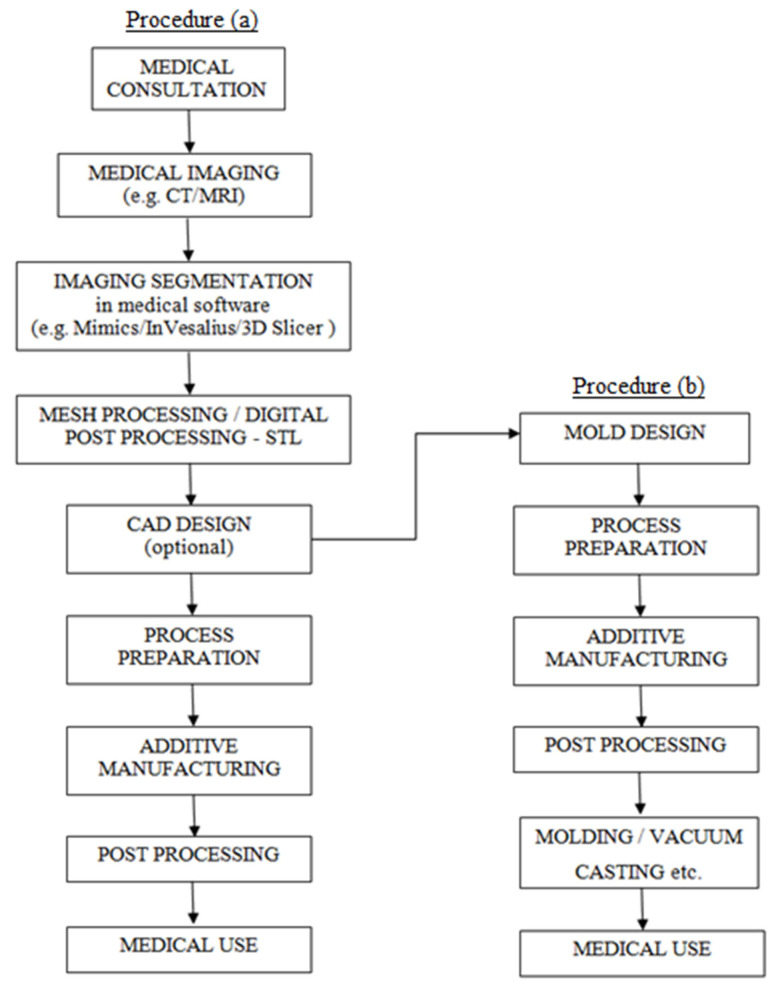
Methodology of producing physical anatomical models.

The methodology for the rapid manufacturing of physical models for medical applications was presented in the literature as early as 2004 by the team of Gibson et al. [18], outlining the most common processes for obtaining the finished product using additive manufacturing, starting with medical imaging. The methodology presented in Figure 3 divides into two paths at the CAD design stage, depending on the model application, the available budget and technology, and the type of imitated tissues: soft or hard.

The process begins with medical consultation and a diagnostic examination using medical imaging (the most commonly used techniques are computed tomography—CT, or magnetic resonance imaging—MRI), which gives an overview of the situation. In cooperation with the surgeon, engineer and radiologist [19], the attending physician decides what character the physical model must have, determines the functionality of the model, its application, and thus considers the budget and the choice of technology.

The medical imaging data are imported to medical programs that enable work on DICOM (Digital Imaging and Communications in Medicine) files, where they are subjected to a segmentation process, as a result of which a digital spatial model is obtained and saved in STL format. The model most often requires a digital post-processing, so its surface meets the requirements imposed by doctors (visibility, functionality) and technological requirements (adaptation to the manufacturing capabilities of the 3D printer and obtaining a waterproof surface) [20,21].

The obtained digital model usually requires implementing corrections such as smoothing, removal of artifacts, surface reconstruction, and closing holes. The next stage concerns the design in CAD programs, but it is not a mandatory element. CAD is most often used in the design of guiding instruments [10], for reconstructions in the craniofacial area, where a template is designed for cutting the bones of the arrow/scapula [6,7,8] or when the parallel process of designing the casting mould is taken for the production of models imitating soft tissues in the casting process [17,22]. The methodology at this stage is divided into two paths, depending on the design assumptions, available funds and manufacturing technology.

Procedure (a) contains the standard steps required to generate the model with additive manufacturing techniques. The digital model, usually stored in the STL format (Standard Triangulation Language, representing triangular mesh of a 3D object) is imported to the dedicated software for individual devices (3D printers), where the process of slicing the model and generating an NC (numerical control) code takes place. The information in the form of a generated program—NC code—is transferred to an additive manufacturing device. This marks the start of a manufacturing process, specific to a selected 3D printing method. The obtained physical model most often requires complex post processing and labour consumption, also depending on the chosen manufacturing method. Usually, it consists of removing support material, grinding and smoothing the surface, cleaning (using alcohol, water, or other agents), curing in UV light (also known as post-curing), and hardening or coating the surface with resins, gluing and many others. The final product, depending on the material used and design assumptions, might be additionally sterilised before medical use, depending on particular requirements.

In parallel, Procedure (b) (Figure 3) appears in the literature, presented, among others, in 2014 by the team of Cheung et al. [23], in 2016 by Adams et al. [22] and in 2017 by Witowski et al. [17] All teams working on low-cost models imitating soft tissues, developed a similar methodology for obtaining multi-material models, partially casted using plastics. At the CAD design stage, the obtained digital model (or at least the external shape, as in example of a kidney) serves as the basis for the design of the casting mould. The design should also include basic elements of the gating system (pouring cup, sprue, overflow, vents, etc.) Moreover, at the mould design stage, the subsequent demoulding process should be taken into account, during which the model cannot be damaged. As in the previous methodology, the obtained digital model of the mould is saved in the STL format and imported to the selected manufacturing device. The printed casting mould undergoes a similar post-processing as the mould mentioned above. The main idea is to get a smooth inner surface. Before the casting mould is assembled, any other previously 3D printed or cast components (e.g., blood vessels, tumours, etc.) should be fixed inside. Then, the prepared form can be poured with the selected material, imitating a specific tissue. The casting process can be performed classically (gravitationally and in open air, which is suitable for low-volume models), or in the presence of a vacuum (vacuum casting) to obtain a homogeneous model without air bubbles that can deform the visibility and affect the properties and functionality of the physical model [16]. After casting, the mould must remain closed for a certain period of time, depending on the setting time of the material. The final step is to open the mould, optionally perform post processing, and then deliver the finished physical model to the medical facility.

Both paths of the process can be undertaken simultaneously because the components of the model (e.g., blood vessels, tumours) can be printed according to the methodology (a), while the outer layer (e.g., kidney cortex) will be obtained in the process (b). It should be made clear that in the case of high-budget solutions, it is possible to obtain models from materials with different properties and hardness, and thus, imitating soft tissues, in the process of direct multi-material 3D printing presented in the methodology (a); an example would be the use of the hi-end PolyJet technology.

## 3. Results

### 3.1. Design

#### 3.1.1. Medical Images and Their Properties

The process of acquiring physical models begins with a medical consultation, as a result of which the patient is referred for diagnostic tests, including medical imaging (most often performed are computed tomography or magnetic resonance imaging).

**Figure 4:**
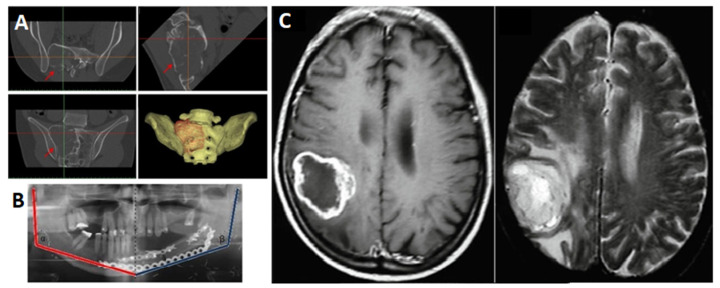
Examples of different use of medical imaging: (**A**) CT images of the pelvis and the generated 3D model [28], (**B**) measurements taken on X-ray medical imaging after mandibular reconstruction surgery [29], (**C**) MRI of the brain [30].

The acquired medical images saved in the internationally known format DICOM will allow opening the files basically all over the world, using all software (open source and commercial alike) dedicated to work with medical imaging. One of the important features of medical imaging, influencing the correct segmentation and design of the model, is slice thickness and slice spacing [24]. Currently, devices such as magnetic resonance imaging (MRI) or computed tomography (CT) allow for obtaining high image resolutions. Thanks to this, even during a routine scanning protocol, values for layer thicknesses from 0.6 mm to 2 mm and voxel sizes from 0.2 mm to 0.6 mm are obtained [24]. Layer thicknesses below 1 mm and voxel isotropy allow minimising the probability of the partial volume effect in the further design stages [25]. Obtaining the lowest possible layer thickness values during imaging gives great opportunities for reliable imitation of the 3D reconstructed organ, while maintaining high dimensional and shape accuracy. However, it should be considered that in the case of computed tomography, the thinner the layer, the higher the level of radiation delivered to the patient’s body. Therefore, when imaging structures with larger and less complex shapes, such as long bones or the pelvis, 2 mm thick layers are sufficient to obtain accurate models. However, in the case of more complex structures, such as a craniofacial surgery’s case, thinner layers, from 0.5 mm to 1mm, are recommended [26]. The selection of imaging is primarily determined by the physician, but magnetic resonance imaging better presents soft tissues, such as blood vessels or the brain, and this translates into better results during segmentation. In the case of bone tissue, computed tomography imaging works better, providing a clear separation of grey tones between calcium-based tissues, such as teeth and bones, and other types of tissues [27]. Examples of the use of medical images in design procedures are shown in Figure 4.

#### 3.1.2. Medical Images Processing

Medical imaging is imported into software that enables image segmentation. Both commercial and open source/free license programs are available on the market. A wide range of available solutions in this area increases the availability of the final product. The most common programs in the literature are:

- Commercial: Mimics (Materialize NV);
- Free: 3D Slicer (The Slicer Community), InVesalius (CTI, Diadema, Brasil), OsiriX (Pixmeo SARL, Bernex, Switzerland).

In the literature, alternative approaches can be also found. Image segmentation can be performed by the use of computational methods (e.g., MATLAB—The MathWorks) and programs self-made by the researchers; however, they are usually used for comparative research on the accuracy of the segmentation process [24].

Image segmentation, which consists of dividing the image by region (region of interest, RoI) with specific, homogeneous properties, allows for generating a spatial model of a specific tissue. In the case of medical imaging, the range of shades of grey in the pixels is determined. Medical imaging is based on the absorption by individual tissues of a varying degree of radiation (CT) or the emission of a radio signal wave (MRI), which is presented in the form of various shades of grey. The Hounsfield scale, which is a quantitative description of radiological density, has been defined for computed tomography. In addition, when selecting segmentation, most programs enable the automatic selection of the range for a specific organ or tissue, which allows for significant facilitation of the process, especially for hard tissues. The segmentation process itself can be performed automatically, semi-automatically and/or manually, depending on the available image extraction methods. The most frequently used segmentation method is global thresholding [25,31,32] due to its greatest availability. However, in some programs, edge detection [33] and region growing options [34] are also possible. The result is a spatial model in the form of a triangular mesh, saved in the STL format.

In the case of neoplastic lesions, the origin of the lesion plays a significant role in its representation on medical imaging. Unpredictability, in the distribution of the lesion, its shape and degree of development significantly hinders the automatic segmentation of such an element. The solution may be deep learning methods, where, by providing the appropriate number of images of changes, the algorithm learns how to correctly recognize and threshold specific cancers. The solution is still being developed, but in the future, it may be an important element in the segmentation process [35,36].

### 3.2. Technologies and Materials

#### 3.2.1. Additive Manufacturing Technologies

Among the available additive manufacturing solutions in the production of medical models for preoperative and training applications, the most commonly used technologies are Stereolithography (SLA), Digital Light Processing (DLP), Fused Filament Fabrication (FFF), Selective Laser Sintering (SLS), PolyJet (Inkjet Printing). The devices used belong to the group of high-budget and low-budget printers (especially FFF, SLA, DLP). Basic information on the layer thickness and the form of the implemented material is presented in the table below (Table 1).

SLA and DLP technologies use materials curable by laser (SLA) or UV light (DLP). The most commonly used material in these methods are resins, also specially dedicated to medical applications. The specificity of manufacturing with this method consists of hardening the material layer with light according to a given shape, and the material used allows for the production of transparent models with a high dimensional accuracy.

SLS and Inkjet Printing technologies are based on production using powdered materials. In both methods, the material is distributed in the working chamber with a given layer thickness, and then it is bonded according to the given geometry. The differences between the technologies lie in the method of powder bonding. In SLS technology, it is the laser beam that sinters the material. In the case of Inkjet Printing technology, the powder is bonded with a liquid binder. The standard material used in the SLS technology is powdered polyamide PA 12, also known as Nylon 12. At the same time, PA11 or PEEE thermoplastics are also available, but they are less often used compared to PA 12. Additionally, the range of materials includes composites with fillers and additives, changing the mechanical parameters of the obtained product. Due to the material’s porosity, obtained by additive manufacturing, models operating in a humid environment require additional post-processing to protect the product. Inkjet Printing technology is based on the production of models using ceramic powders. Moreover, it is possible to produce multicoloured objects (ColorJet Printing) due to the design of the nozzles containing the liquid binding binder. The construction of the nozzles is based on a classic set of toners, similar to that used in inkjet printers, and is based on four basic colours: CMYK.

Polyjet Printing technology uses liquid photopolymer materials, which are deposited in the form of drops on the working platform, according to a given geometry, and cured with UV light. After exposure, the process is repeated, and another layer of material is applied until a complete product is obtained. In addition, there are extended versions of this technology: Polyjet Matrix and Triple-Jetting. Both solutions rely on the multiplication of the printing nozzle, thanks to which, during one 3D printing process, it is possible to apply different materials with different properties. In this way, the possibilities of producing medical models are increased, consisting of materials of differing hardness and flexibility, and in many colours. The most commonly used materials in the Polyjet technology are light-curing resins. A wide range of coloured, transparent materials, imitating polypropylene, elastic rubber-like materials, as well as biocompatible materials suitable for use in medicine, are available. For Stratasys devices, an additional material solution is available: Digital Material. They are composite materials made by mixing two or more basic materials. The process makes it possible to obtain products of various hardness on the Shore scale, and in a variety of colours. Currently, Polyjet technology is one of the most precise and versatile additive manufacturing technologies, but it belongs to the group of high-budget devices.

Fused Filament Fabrication (FFF) technology is the most widely used additive manufacturing technology. The RepRap project, based on the open design license initiated in 2005, contributed to this. As a result, low-cost devices began to be used on a large scale, constantly developing the possibilities of technology and the available range of materials. The FDM/FFF technology is based on the deposition of plasticised plastic by nozzles on the work platform, which moves along the OZ axis by the thickness of the layer until the finished model is obtained. The most commonly used materials are PLA, ABS, and Nylon. However, the range of materials includes many more examples: alcohol or water-soluble PS/HIPS, highly flexible materials (TPU) or materials with additives and fillers such as wood, copper etc., or experimental materials for medical applications. Due to the high availability of low-budget and professional industrial devices, the possibility of obtaining functional medical models at relatively low costs has significantly expanded [37].

Various additive manufacturing technologies are available, and the market is still growing rapidly. Technologies using metal powders such as Direct Metal Laser Sintering (DMLS), Selective Deposition Lamination (SDL), Binding Jetting, and Electron Beam Melting (EBM) in medicine, are used primarily in implantology [28]. The metal materials used, such as titanium powders, are biocompatible and bioactive, which makes it possible to use them in clinical conditions. The manufactured models are usually individualized implants, adapted to the patient’s individual bone geometry. In the case of anatomical models, the above technologies are not used, therefore they will not be considered in the further part of the review.

#### 3.2.2. Non-Additive and Supporting Manufacturing Processes

Medical models can be manufactured during one 3D printing process or divided into several stages, where individual components are produced on different devices and using different production methods. In the literature, the most common supplementary method is casting; this is both with the use of vacuum casting devices and classic casting with plastics [12,16,17,22,23,27,38,39]. Some works utilised the devices using a vacuum, when it is required to get rid of air bubbles that may disturb the visibility of the structures inside the produced model [16]. Manual casting can be carried out in various ways, both through the material in layers on the master model [38], applying the material using a syringe [40], and the classic pouring into the mould [41]. The most commonly used materials for the casting process in the case of medical models are silicones and very low hardness resins (Shore 00/Shore A scale) [27]. Thanks to the use of the plastic casting method in the model manufacturing process, it is possible to obtain an imitation of soft tissues and obtain a much lower hardness of the model than in the case of most classic methods of additive manufacturing; at the same time, there are low production costs [27].

In most cases presented in the literature, the procedure for producing a casting mould takes place in two ways. The first is to produce a mould designed from the negative of the organ, with the use of additive manufacturing [27]. The second method is 3D printing a model from harder materials and then creating the negative of the mould by casting [12]. The obtained mould may be additionally post-processed by smoothing the negative of the mould (removing the layering effect) and covering the negative with a thin layer of material, facilitating the separation of the model from the mould at the time of demoulding.

#### 3.2.3. Materials and Their Properties

Materials for the production of models imitating soft tissues are selected on the basis of the assessment of living tissues on several levels. The most common method is the evaluation of material samples by doctors. Surgeons working with specific tissues on a daily basis, based on knowledge and experience, and based on haptic assessment, select the most appropriate material [16]. In addition, cutting and suturing procedures can be performed on material samples by a qualified medical team or by medical students [41]. Another form of collecting information on the hardness or stiffness of soft living tissue is the assessment of animal organs and a comparison based on the tactile perception or objective measurements [22]. The evaluation of materials and tissues can also be made based on measuring systems. However, the parameters for individual tissues may differ due to the existing variables. The hardness and properties of the tissue are influenced by the origin of the organ (human or animal), the patient’s age, sex, lifestyle, measurement on a living person or on a cadaver, and measurement outside or inside the body, etc. [16].

A. N. Blanco et al., on the basis of the information contained in the literature on liver stiffness [16], selected a narrow group of materials used in the model manufacturing process. The average stiffness of a healthy liver is 5.49 kPa, which is approximately 52 Shore 000 or 25 Shore 00. Due to the lack of equivalence between the Shore 00 and Shore A scales in the lower ranges, the results were supported by a medical interview. Based on the obtained data, it was possible to select adequate materials imitating the tissue. The research mentioned that, despite reducing the hardness of the rubber, especially polyurethane rubber, the obtained value is slightly higher, limiting the possibility of an adequate tissue imitation. However, these are materials with good transparency, which may compensate for differences in the hardness of the phantom. The finally selected materials were characterised by three levels of hardness:

- Eco Flex 00-30, SmoothOn with a hardness of 30 Shore 00, a working life of 45 min and a setting time of 4 h,
- SORTA-Clear, SmoothOn with a hardness of 18 Shore A, a service life of 60 min,
- Clear Flex 30, SmoothOn with a hardness of 30 Shore A, low pot life [16].

C. L. Cheung et al. [23] determined the selection of flexibility and mechanical properties of the kidney and pelvis, based on medical consultation and assessment. Material samples, i.e., Dragon Skin 30 silicone, Shore A 30 SmoothOn and Slacker Tactile Mutator, SmoothOn, were mixed in various proportions and then presented to a team of urologists who made a selection based on their own experience. The research emphasised that in the case of training models, the correct imitation of the mechanical properties of the tissue is of particular importance. Incorrect tissue imitation may lead to training participants obtaining incorrect feedback generated by the model, which may, in the future, translate into generating too much pressure on the living tissue, thus increasing the risk of causing organ damage.

The team of H. Riedle et al. [11] divided the assessment of the selected materials into four main properties of hardness, stiffness, flexibility and surface roughness, which were evaluated by a team of expert surgeons compared to a living heart. The material selected was 20 Shore A hardness silicone (ACEO Silicone GP Shore A 20). The measurement procedure was performed using a seven-step bipolar scale. As a result, only the silicone material that imitates the major blood vessels was classified as realistic in terms of the hardness and stiffness of the material. In the case of heart valves, it has been suggested that the selected material is clearly too hard and too stiff, which may result in an unrealistic tissue thickness due to technological limitations (minimum 1 mm). The silicone material with a hardness of 20 Shore A was found to be generally too stretchable, and the surface was rough (layering effect due to the rapid manufacturing method) compared to a living organ. Quantitative analysis was not performed, as it was based on the parameters available in the technical datasheet.

#### 3.2.4. Examples of Used Materials and Technologies

The materials used by researchers were characterised by different levels of hardness, stiffness and individual properties. They are closely related to the technology used and the manufacturing methodology being implemented. Table 2 and Table 3 present exemplary material and technological solutions, as well as the application of the produced anatomical models, imitating both soft and hard tissues.

### 3.3. Assessment and Application

#### 3.3.1. Medical Models Assessment Techniques

**Figure 5:**
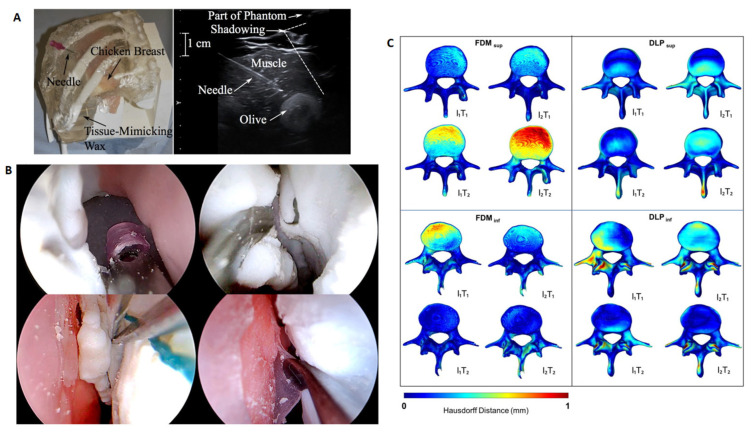
Examples of the assessments: (**A**) 3D printed ribs model with a chicken breast and biopsy needle and ultrasound scan of model [65] (**B**) 3D printed training tool for simulated endoscopic sinus surgery [66], (**C**) comparative analysis of the digital model and 3D scan of the printed model [67].

Depending on the purpose of the research, the evaluation of the obtained models may take the following forms: parametric (objective) and based on experience, and interview (subjective). Parametric evaluation is most often performed with the use of measuring and diagnostic equipment, such as medical imaging such as MRI, CT or ultrasonography, or with the use of medical equipment, such as an endoscope (Figure 5).

F. Adams et al. [22] assessed the acquired models using three diagnostic solutions: computed tomography, ultrasonography and endoscopic examination. In the case of using CT assessment, the aim was to determine the shape-dimensional accuracy in relation to the base model, which was the digital model of the human kidney, also obtained in the CT examination. In that case, the error was negligible (according to the researchers) for one of the materials—silicone elastomer (Ecoflex)—while for the others (agarose gel and PDMS), it was ~0.6 mm. Moreover, the CT evaluation revealed the correct reconstruction of the morphological details of the collecting system in the casting process, and the obtained information allowed for the validation of the model thus produced for use in training on the use of the endoscope and performing tests [22]. The use of computed tomography allows the determination, first of all, of the dimensional and shape accuracy obtained in the process of additive manufacturing and/or the accuracy of the model after preoperative sterilisation [6]. An alternative method of assessing the shape and dimensional accuracy is to perform a spatial scan using a 3D scanner [8].

The ultrasonography assessment is most often aimed at validating materials in terms of the acoustic impedance of the ultrasonic wave. The phantoms subjected to such assessment are used in training in the handling and working with an ultrasound scanner, as well as in simulating the examination process during the training of operating and treatment procedures [11,22].

The endoscope is used to evaluate the model in terms of the possibility of conducting training in the performance of procedures that require operating the endoscope. The surface of the structures examined inside the model and their quality are assessed in relation to the endoscopic image obtained during the examination of a living organ, as well as the level of imitation of the internal structures of the organ [22].

The method of validating the obtained model and the level of imitation of tissues is also performing a simulated operation. Treatment procedures are performed on a model, usually embedded in conditions similar to real ones. C. L. Cheung et al. [23] handed over the obtained model in the form of a trainer to a group of four experts urologists, who performed a simulated procedure. The specialists assessed the model in terms of aesthetics, prototype handling and overall impression. The information obtained in the interview after the surgery allowed the improvement and increase in the quality of the manufactured trainer [23].

Nevertheless, the most common assessment of the models is the medical assessment by physicians. Usually, a team of specialists receives a questionnaire, which is filled in based on the aesthetic and tactile impressions and, possibly, experiences obtained in the process of a simulated operation. Based on professional experience, they are able to determine the reliability of imitated tissues, the behaviour of the model during a simulated procedure, or its suitability in the process of planning the operation. The obtained information increases the quality of the anatomical models and improves their properties [40,46,68,69,70]. Such an assessment can also be made by patients who determine the level of understanding of the operated organ’s anatomy, physiology, pathology characteristics, and planned operating procedures, comparing the knowledge obtained with and without the 3D printed model [45,71].

#### 3.3.2. Applications of Fabricated Models

**Figure 6:**
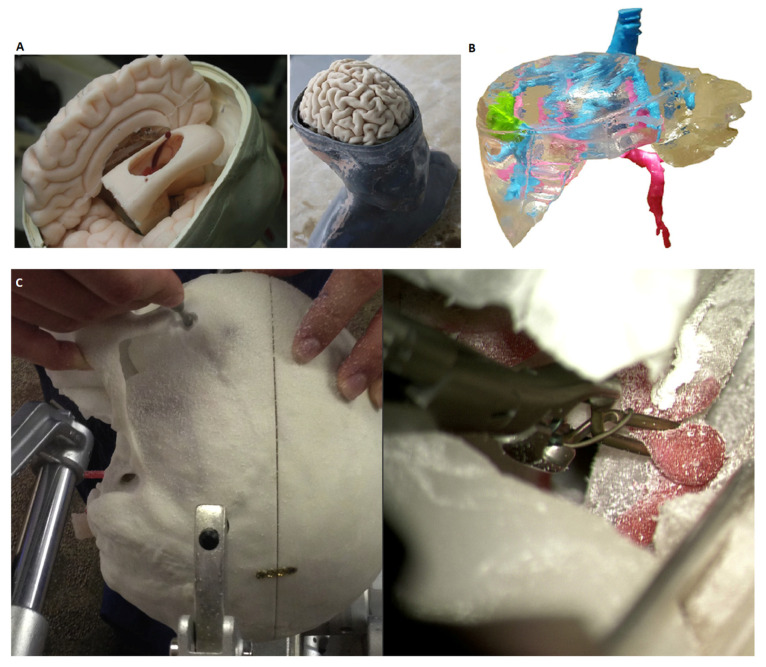
Examples of the use of physical models in medicine: (**A**) Endoscopic third ventriculostomy surgical simulator [73], (**B**) model for liver preoperative planning [17], (**C**) simulated surgery on the skull [47].

Anatomical models are used in medical practice in many areas (Figure 6). Among the most important, it is worth mentioning pre-and intraoperative support, namely, the possibility of performing a surgical procedure under controlled conditions (known as the simulated surgery [72]). Moreover, the models are an excellent tool for training in the field of surgical procedures or learning about specific types of pathology at the level of patient and family education, or at the level of less experienced trainees and medical students. Indeed, 3D printing is also used to produce personalised guiders, supplementing the basic surgical instruments. Due to the relatively low cost of producing anatomical models, especially with the use of low-cost 3D printing technologies, the use of anatomical models at various stages of medical practice is possible and easily accessible.

Pre- and intraoperative support is particularly well presented by the mandible models used to prepare for bone reconstruction surgery. The procedure is based on the excision of a part of the lower jaw damaged as a result of the disease, and the reconstruction is performed using the patient’s arrow bone or scapula. The study, led by T. P. Farias et al. [F11], presented the use of a 3D printed model of the jaw in the process of the detailed planning of the treatment procedure. Titanium plates were fitted and placed on the anatomical model of the bone. Then, test cuts were made on the models of the mandible and the bone used for reconstruction.

Indeed, 3D printing is also used to educate students and during training courses dedicated to specialist doctors. By combining the available tools used in medicine training and additively produced models, W. Clifton et al. [62] obtained a trainer that responds to the specific demand (i.e., not commercially available). The lack of simulators typically designed for advanced cervical spine surgery tools prompted the team to develop a simulator that allows for the manual placement of screws on the C2 section. The use of 3D printing allowed not only the creation of vertebrae, requiring a surgical procedure, but also, due to the specificity of the technology, the corticocancellous interface to be obtained.

Performing a simulated operation on a model produced with rapid manufacturing methods, combined with the casting procedure, was also implemented by the team of C. L. Cheung et al. [23], which created a trainer used for training in paediatric laparoscopy. The produced model of the kidney, with the use of silicones, consisted of the kidney cortex, dilated renal pelvis, ureter and overlying peritoneum, simulating the traditional transmesenteric approach. The model has been scaled to a size similar to a 10-month-old baby. In the production of the training model, a methodology combining both classic additive manufacturing (Inkjet Printing) and manual casting, with the use of silicones, was used.

In addition to the pre-operative preparation of doctors, anatomical models can also be used to prepare the patient. J.C. Bernhard et al. [45] assessed the patient’s understanding of the kidney’s anatomy, physiology, pathology and surgical procedures. The model of the kidney, made of a transparent photopolymer, additionally contained stained blood vessels and a tumour in a different colour. Due to the transparency, the relations between individual structures were visible. Thanks to the presentation of the kidney 3D model with the tumour, the patient’s understanding grew in each area studied.

The presented solutions are examples of applications of anatomical models in medical practice. However, it is worth bearing in mind that the area of application is much larger and depends on the financial capabilities of the team, the requirements for models, the needs of the patient or doctors, and the general anatomy of the patient.

## 4. Discussion

Based on the literature review, the most common application of physical models can be expressed in four areas: education, presurgical planning, simulated operation and surgical training. The remaining areas are guiders or surgical instruments, research to improve the accuracy of the model and their assessment, and in the case of hard tissue, models dedicated to the reconstruction of the mandible and intraoperative navigation. It is important to bear in mind that, usually, one model is used in more than one area. The functionality of the models is dedicated to medical requirements, but when there are no destructive tests, the model can be used multiple times (e.g., for educational purposes).

**Figure 7:**
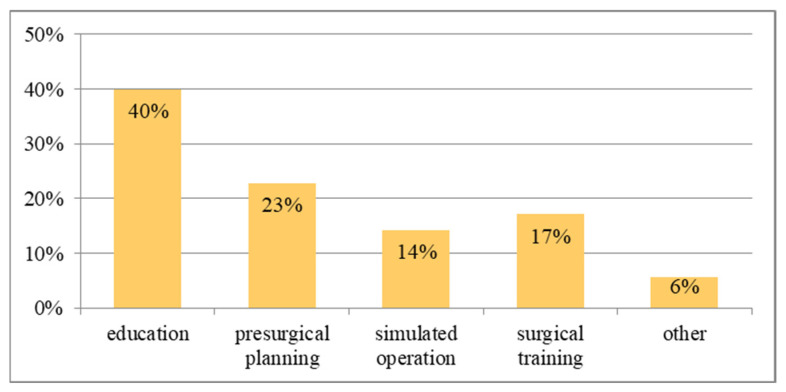
The percentage distribution of applications of physical models for soft tissues.

In the analysed literature, for soft tissue models, 40% of them were used for educational applications and 23% for preoperative planning (Figure 7). At a similar level, 17% and 14%, models imitating soft tissues are used in operational training and as part of simulated surgery. The most common combination of areas was education and operational training.

**Figure 8:**
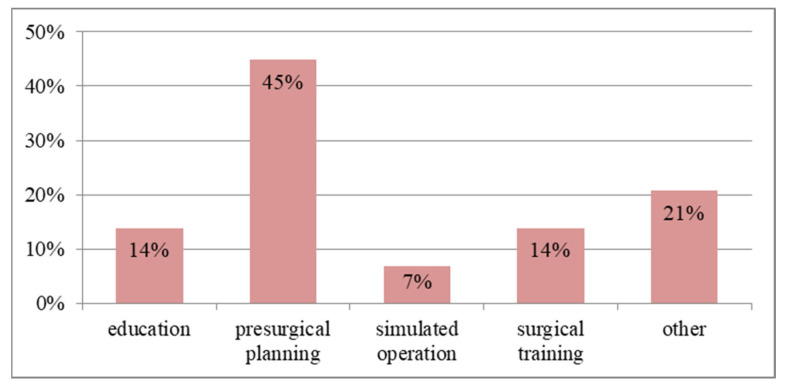
The percentage distribution of applications of physical models for hard tissues.

The application distribution for the physical models of hard tissue is illustrated in Figure 8. It is clear that 45% of the models were used in the presurgical planning procedure. Models of the lower jaw were used particularly often in the procedure of preparing the reconstruction of the craniofacial bone. The hard tissue models were used much less frequently in the education process compared to soft tissues, as only 14% of model applications were used this way. A significant percentage of uses occurred with other options. Among them, intraoperative navigation is worth mentioning, as it simplifies the surgery and increases the patient’s safety and the doctor’s confidence.

Education concerns both doctors, both more and less experienced, medical students, preparing for the profession of a doctor, physiotherapists, dentists, etc., as well as patients and their families. The purpose of the models is, above all, to reliably reflect the relationship between anatomical structures and pathology, as well as illustrate the potential results of the planned surgery. Therefore, shape and dimensional accuracy, especially at the level of the arrangement of elements making up the model, and transparency (especially for soft tissues), are important. Tissue imitation for educational purposes is less important, unless the education is combined with training in the performance of surgical procedures.

Presurgical planning includes familiarising oneself with the lesion and its location, planning the surgical approach, considering various operating scenarios, matching implants and developing a reconstruction procedure. In the case of hard tissues, it is important to maintain shape and dimensional accuracy, especially in the reconstructed area undergoing surgery. For soft tissues, transparency of a model is a great advantage, which complements the preparation for surgery based on medical imaging. The transparency of the model facilitates the recognition of the area undergoing surgery and enables the planning of various treatment scenarios. Tissue imitation at this stage is not essential, unless the presurgery preparation includes a simulated operation procedure.

The simulated operation is a kind of training, during which the doctor performs all the operating procedures on a physical anatomical model. Prepared operating scenarios during preoperative planning can be implemented on an anatomical model, thanks to which the procedure on the patient will be safer, and the time of the operation will be reduced. At this stage, various attempts can be tested to remove the lesion or practice the reconstruction procedure and estimate the risk of surgery. Therefore, physical models should, first of all, reliably imitate the tissues to faithfully simulate the patient’s actual condition. The shape and dimensional accuracy are equally important at this stage. The transparency of the model will be of added value, but it is not necessary.

Surgical training is a combination of all the above-mentioned functions of the model. It combines elements of preoperative preparation, i.e., getting to know the change and anatomy of a diseased organ. The model performs an educational and practical function at the anatomical level, i.e., getting to know the individual case. Doctors using the model can perform medical procedures on the model and thus learn about the procedures for dealing with unconventional cases. Additively manufactured anatomical models can present very different cases from the standard, which is not achievable with traditional commercial models. Training models can be used not only to perform a simulated operation, but also constitute a tool for practising diagnostic procedures, e.g., ultrasonography or endoscopic examination. Requirements for all model applications are presented in summary Table 4.

## 5. Conclusions

Additive manufacturing methods make it possible to obtain models of complex shapes, often impossible to achieve with the use of more traditional manufacturing methods (e.g., machining). Thanks to this, it is possible to produce models with high shape and dimensional accuracy, based on the actual and individual anatomy of the patient’s organ. The growing availability of 3D printing methods, not only in the area of the industry, but also for individual applications, has facilitated and increased access to the acquisition of functional anatomical models at low costs. In addition, wide access to software based on open-source licenses, allowing for work with medical imaging and image segmentation, has influenced the popularisation of the use of models imitating both hard and soft tissues. The searched solutions for combining production methods, and the continuous evolution of the methodology of manufacturing such models ensure development in this area. An important stage at the moment is obtaining the correct combination of materials with precisely defined parameters, which will ensure the correct imitation of soft tissues; this will affect the correct feedback for doctors at various stages of planning surgery or training. In addition, it is important to standardise the assessment procedure of such models, both in terms of material and technology.

Physical anatomical models are widely used in the medical practice, and they allow an improvement in the quality of treatment and patient safety by planning and carrying out operations simulated on a phantom. They facilitate familiarisation with the relationship between anatomical structures and diseased or otherwise damaged tissues. Moreover, they are a very good tool for training and educating students, doctors, patients and their families. Medical models are becoming an increasingly frequent element of the pre-operative preparation procedure, and may become a standard in diagnostics and treatment in the future.

Currently, research is moving towards the standardisation of model manufacturing procedures at all stages, from design to evaluation. In addition to the selection of materials and technology, which will enable the imitation of tissues at a level close to reality, standardised evaluation procedures are needed. The assessment of the selected materials, especially those imitating soft tissues, and the overall functionality of the model, are still based primarily on the subjective feelings of doctors. Therefore, the challenge is to define the necessary conditions that must be met by the model so that the assessment procedure is as objective as possible, and so it ultimately excludes intermediaries from the process. In this way, it will be possible to provide the surgeons with a functional and compliant physical model in a shorter time. Thus, doctors’ time will be saved, and the procedure of obtaining the finished product will be improved. Such a step will affect the wider access to medical models, which in the future may constitute an integral part of pre-operative preparation or medical education at various levels. In addition, to increase the availability of models, it is important to take into account the minimisation of costs, which translates into the materials and technologies used in the manufacturing process.

The development of the following steps to complement the existing procedure methodology is, therefore, an important step in popularising the use of rapid manufactured anatomical models.

## Figures and Tables

**Table 1:** Basic information about rapid manufacturing technologies.

Technology	Examples of Printers	Layer Thickness [mm]	Form of Material	Used Materials
Fused Filament Fabrication (FFF)	**LOW-COST**	0.10–0.33	Filament spool	ABS, PLA, HIPS, PP, TPU, Nylon
	MakeBot, Stratasys Ultimaker Prusa Ender Zortax			
	**PROFESSIONAL**			
	Vshaper Rise 3D Dimension 1200 es			
Stereolithography (SLA)	**LOW COST**	0.05–0.15	Liquid photopolymer	Resins: standard, pure, casting, with increased strength, high temperature, dental, rubber-like
	Form+, Formlabs, Startasys Creality Anycubic The Nobel, XYZ			
	**PROFESSIONAL**			
	Exosys Projet, 3D Systems			
Selective Laser Sintering (SLS)	EOS Eosint P395, EOS Lisa, Sinterit	0.060–0.150	Polymer powder	PA (12, 11), PS, TPE, PP, PEEK, Nylon
Inkjet Printing	zPrinter 450, 3D Systems Spectrum Z510, 3D Systems zPrinter 310+, 3D Systems	0.1	Ceramic powder	Plaster (CaSO_4_)
Polyjet Printing	Objet30Pro, Stratasys Objet 500 Connex3, Stratasys Objet260 Dental Selection, Stratasys	0.016–0.028	Liquid photopolymer	Resins: standard, flexible, simulating PP or ABS, high temperature, transparent, medical

**Table 2:** Examples of materials, technologies and applications of models imitating soft tissues.

S. No	Authors	Year	Discipline	Materials	Technology	Organ	Use
1	R. A. Watson [42]	2014	Hepatology	Nylon	Selective Laser Sintering	Portal and hepatic veins	Surgical education
2	Y. Zheng et al. [43]	2016	Hepatology	ABS	Objet 500 Connex 3 (Polyjet)	Pancreas Artery Portal vein Spleen	Surgical education Preoperative planning
3	J. S. Witowski et al. [17]	2017	Hepatology	PLA Silicone rubber—Polastosil M-2000	Ultimaker 2+ (FFF) cast + internal structures Manual casting	Liver Internal structures Tumour	Preoperative planning
4	A.M. Blanco et al. [16]	2018	Hepatology	PLA Shenzhen Esun Industrial Co./Colorfila PVA Shenzhen Esun Industrial Co.—support material Silicone rubbers: The Smooth-On EcoFlex 00-30 SortaCLEAR Shore A 18 ClearFlex 30 by SmoothOn	Sigma BCN3D (FFF)—cast + internal structures Manual casting (material a)) Renishaw Vacuum System 5/01(material b)) Manual casting (material c))	Liver Internal structures Tumours	Presurgical planning
5	C. L. Cheung et al. [23]	2014	Urology	Powder ZP-131 + bonding agent ZB-60 Infiltration process Z-Bond 90 Silicon rubber—Dragon Skin 30 + Slacker Tactire Mutator (SmothOn)	Spectrum Z510 3D Printer (Inkjet Printing)—cast Manual casting + vacuum chamber (degassed process)	Kidney Dilated renal pelvis Ureter Overlying peritoneum	Training models for paediatric laparoscopic pyeloplasty
6	JC. Bernhard et al. [44]	2015	Urology	Photopolymer	Objet 500 Connex 3 (Polyjet)	Kidney Tumour Internal structures	Patient education
7	F. Adams et al. [22]	2016	Urology	Engineered wax material Photopolymer VeroClear Silicone rubber—The SmoothOn Ecoflex 00-20 Agarose gel—Agarose Electran Polydimethylsiloxane (PDMS)	3Z Pro, Solidscape (high precision 3d printing)—inner wax mould Objet 260 Connex 3 (Polyjet)—external cast Manual casting	Kidney	Presurgical planning Simulated operation Endoscopy training
8	H. Lee et al. [45]	2018	Urology	Photopolymer	Objet 260 Connex 3 (Polyjet)	Kidney Tumour	Presurgical planning Students’ education
9	H. Riedle et al. [11]	2020	Cardiology	ACEO^®^ Silicone GP Shore A 20	ACEO^®^ Technology (drop-on-demand 3D printing)	Heart Aorta	Simulated operation
10	S. R. de Galarreta [12]	2013	Cardiology	Full-cure 720 Silicone rubber—SLM VTX 950 WA–70 wax Resin—SLM PUR	Objet Eden 330 (Polyjet)—master model Manual casting MCP 4/01 Vacuum Casting Machine	Abdominal Aortic Aneurysm	Validation deformation and optical methods
11	I. O. Torres et al. [46]	2017	Cardiology	Polyjet Material Rubber FLX930 Polyjet Material Standard Plastic RGD810 Polyjet Digital Material Tango Plus + Vero Clear Shoe 60 Flexible Photopolymer Resin for Form1+ MakerBot Tough PLA	Objet 350 Connex 3 (Polyjet) Formlabs Form1+ (SLA) Makerbot (FFF)	Abdominal Aortic Aneurysm	Simulated operations Training models
12	T. Mashiko et al. [38]	2015	Neurology	ABS M8012 from Asahi Kasei-Wacker Silicone (moulding silicone)	UP!Plus 3D Printer (FFF) Manual coating	Cerebral aneurysm	Surgical training Simulated operation
13	J. Ryan et al. [47]	2015	Neurology	Gypsum powder ABS Casting silicone—The Smooth-On Mould-Max 60 Hydrogel (gelatine + agar gel powder)	zPrinter 350 (Inkjet Printing) Stratasys Dimension 1200es (FFF) Manual casting	Skull Anterior horns Brain	Surgical training Students’ education
14	J.P. Thawani et al. [48]	2016	Neurology	Polycarbonate-like photoreactive polymer	ProJet 6000 3D Printer (SLA)	Arteriovenous Malformation	Presurgical planning Surgical training Education
15	J. R. Ryan et al. [49]	2016	Neurology	Photopolymer Shore A 27 ABS Silicone rubber—The SmoothOn Mold Star Silicone Rubber—DragonSkin + Slacker Tactile Mutator (The SmothOn) Composite Material	Objet 500 Connex 3 (Polyjet) Stratasys Dimension 1200 (FFF) zPrinter 650 (Inkjet Printing)	Vascular Brain Skull	Surgical training Presurgical planning
16	W. Mussi et al. [27]	2020	Neurology	PLA PLA wood-loaded Silicone rubber—The SmoothOn EcoFlex 00-50 Silicone rubber—DragonSkin 10	MakerBot Replicator 2X (FFF) Manual casting	Skull Brain Tumour Tentorium Flax	Simulated operations Training models
17	S. Bustamante et al. [39]	2014	Pulmonology	Photosensitive flexible liquid resin	Object 350 Connex3 (Polyjet)	Tracheobronchial tree	Anaesthesia education
18	S.N. Kurenov et al. [50]	2015	Pulmonology	TangoPlus (Thermoplastic elastomer)	PolyJet Eden 260 V (Polyjet) Objet 500 Connex 3 (Polyjet)	Pulmonary arteries	Presurgical planning Device development Anatomy study
19	J. T. Lichtenstein et al. [51]	2016	Ophthalmology	PA2200 Silicone mixture—VTV 800 (SLM Solution) + VTN 4500 (The SmoothOn)	Selective Laser Sintering (bone + moulds) Manual casting	Globe Nerve Muscles Lids Bone	Surgical training Education
20	R. Javan et al. [52]	2016	Orthopaedics	Rubber-like material Platinum-cure silicone gel -Ecoflex 00- 50; Smooth-On High-detail polyamide Highly concentrated gelatine solution	Zcorp 3D printer (Inkjet Printing) Manual casting	Spinal cord Nerve roots Intervertebral discs	Surgical training Students’ education

**Table 3:** Examples of materials, technologies and applications of models imitating hard tissues.

**S. No**	**Authors**	**Year**	**Discipline**	**Materials**	**Technology**	**Organ**	**Use**
1	M.D. Tam et al. [53]	2012	Orthopaedics	Plaster powder	zPrinter 450 (Inkjet Printing)	Scapular osteochondroma	Presurgical planning
2	Y.Gan et al. [54]	2015	Orthopaedics	Acrylate resin—Somos 14120	Stereolithography (SLA)	Surgical guiders: tibia and femur	Intraoperative navigation
3	D. Pacione et al. [4]	2016	Orthopaedics	VeroWhite, VeroMagenta VeroBlack	Objet260 Dental Selection (Polyjet)	Skull Vertebras Vessels Metal parts	Presurgical planning
4	R. Javan et al. [52]	2016	Orthopaedics	Gypsum (contains calcium)	Zcorp 3D printer (Inkjet Printing)	Vertebras (lumbar region)	Surgical training Students’ education
5	J. P. Guenette et al. [55]	2016	Orthopaedics	Objet RGD525 High Temperature Vero White	Objet 500 Connex3 (Polyjet)	Vertebras	Presurgical planning
6	M. Putzier et al. [56]	2017	Orthopaedics	PA2200	EOS Eosint P395 (SLS)	Vertebra Guider for pedicle screw placement	Presurgical planning Intraoperative navigation
7	L. Weigelt et al. [57]	2017	Orthopaedics	PA2200	Selective Laser Sintering (SLS)	Surgical guiders for bones: tibia/fibula	Presurgical planning Surgical guiders
8	H. J. Park [58]	2018	Orthopaedics	Polypropylene	Stratasys Objet30Pro (Polyjet)	Spine (lumbar vertebrae)	Surgical training Students’ education
9	L. Piles et al. [59]	2019	Orthopaedics	Sakarat ABS-E	XYZPrinting DaVinci 1.0 (FFF)	Scapula Humorous	Presurgical planning
10	L. Frizziero et al. [60]	2019	Orthopaedics	PLA	EZT3D Delta (FFF)	Bone (femur)	Presurgical planning Preoperative diagnosis
11	W. Clifton et al. [61]	2019	Orthopaedics	PLA Melted 10% ballistics gel	Ultimaker S5 (FFF)	Vertebras	Surgical training Students’ education
12	A. Mishra et al. [62]	2019	Orthopaedics	PLA	FFF	Pelvis Hip Spine Knee Shoulder Elbow Wrist joint	Presurgical planning
13	T. P. Farias et al. [63]	2013	Cranio-Maxillofacial Surgery	Composite of gypsum, cyanoacrylate, and ZP150	Z510 (Inkjet Printing)	Mandibular Iliac crest Fibula	Presurgical planning Simulated operation
14	A. Masaki et al. [64]	2014	Cranio-Maxillofacial Surgery	Plaster powder	zPrinter 310+ (Inkjet Printing)	Mandibular	Presurgical planning
15	S. K. Malyala et al. [7]	2016	Cranio-Maxillofacial Surgery	PLA	MakerPi M14 (FFF)	Maxilla Mandibular Preliminary ver. of the implant	Presurgical planning Simulated operation
16	L. Ganry et al. [6]	2017	Cranio-Maxillofacial Surgery	Polyamide 12 (poly-lauroctam)	Selective Laser Sintering (SLS)	Mandibular	Surgical guides for free flap mandibular reconstruction Model of reconstructed mandibular
17	S.M. Werz et al. [40]	2018	Cranio-Maxillofacial Surgery	ABS (MakerBot Industries) PLA(MakerBot Industries) Silicone rubber (NEUKASIL RTV 23/Crossliker A7	MakerBot Replicator 5th Generation (FFF) Manual applied silicone with syringe	Upper jaw Lower jaw	Training models for oral and maxillofacial surgery
18	F. Górski et al. [8]	2019	Cranio-Maxillofacial Surgery	ABS	Stratasys Dimension 1200 (FFF) MakerBot Replicator 2X (FFF)	Lower jaw	Presurgical planning
19	S. Chen et al. [5]	2017	Anatomy	PLA	Ultimaker 2 (FFF)	Skull	Students’ education
20	C.S. Favero et al. [9]	2017	Orthodontics	Photopolymer resin FLGPGR02	Form 2 printer (SLA), Juell 3D (DLP), Objet Eden260V Dental Advantage (Polyjet), large-frame Vector 3SP (3SP), Perfactory Desktop Vida(DLP)	Maxillary arch	Assessment of the accuracy of orthodontic models

**Table 4:** Requirement features of the models for selected applications.

	Model Features	Shape and Dimensional Accuracy	Transparency	Tissue Imitation
Application				
Education		necessary	necessary	not necessary
Presurgical planning		necessary	added value	not necessary
Simulated operation		necessary	added value	necessary
Surgical training		added value	added value	added value

## Data Availability

Not applicable.
